# Systemic Inflammation/Nutritional Status Scores Are Prognostic but Not Predictive in Metastatic Non-Small-Cell Lung Cancer Treated with First-Line Immune Checkpoint Inhibitors

**DOI:** 10.3390/ijms24043618

**Published:** 2023-02-10

**Authors:** Cédric Mahiat, Benoît Bihin, Fabrice Duplaquet, Claudia Stanciu Pop, Michael Dupont, Thierry Vander Borght, Benoît Rondelet, Jean Vanderick, Bénédicte André, Lionel Pirard, Sebahat Ocak

**Affiliations:** 1 Pneumology Division, CHU UCL Namur (Godinne Site), Université Catholique de Louvain (UCLouvain), Avenue Docteur G. Thérasse 1, 5530 Yvoir, Belgium; 2 Scientific Support Unit, CHU UCL Namur (Godinne Site), UCLouvain, Avenue Docteur G. Thérasse 1, 5530 Yvoir, Belgium; 3 Pathology Department, CHU UCL Namur (Godinne Site), UCLouvain, Avenue Docteur G. Thérasse 1, 5530 Yvoir, Belgium; 4 Radiology Division, CHU UCL Namur (Godinne Site), UCLouvain, Avenue Docteur G. Thérasse 1, 5530 Yvoir, Belgium; 5 Nuclear Medicine Division, CHU UCL Namur (Godinne Site), UCLouvain, Avenue Docteur G. Thérasse 1, 5530 Yvoir, Belgium; 6 Thoracic Surgery Department, CHU UCL Namur (Godinne Site), UCLouvain, Avenue Docteur G. Thérasse 1, 5530 Yvoir, Belgium; 7 Radiation Therapy Department, CHU UCL Namur (Sainte Elisabeth Site), UCLouvain, Place Louise Godin 15, 5000 Namur, Belgium; 8 Pole of Pneumology, ENT, and Dermatology (PNEU), Institut de Recherche Expérimentale et Clinique (IREC), UCLouvain, Avenue Hippocrate 10, 1200 Brussels, Belgium

**Keywords:** immune checkpoint inhibitor, immunotherapy, non-small-cell lung cancer, nutritional status, PD-(L)1 inhibitor, systemic inflammation

## Abstract

Biomarkers of systemic inflammation/nutritional status have been associated with outcomes in advanced-stage non-small-cell lung cancer (NSCLC) treated with immune checkpoint inhibitors (ICIs). However, most of them were not tested in cohorts of patients treated with ICIs in combination with chemotherapy (CT) (ICI + CT) or with CT alone, making it impossible to discriminate a predictive from a prognostic effect. We conducted a single-center retrospective study to search for associations between various baseline biomarkers/scores that reflected the systemic inflammation/nutritional status (Lung Immune Prognostic Index, Modified Lung Immune Prognostic Index, Scottish Inflammatory Prognostic Score, Advanced Lung Cancer Inflammation Index, EPSILoN, Prognostic Nutritional Index, Systemic Immune-Inflammation Index, Gustave Roussy Immune Score, Royal Marsden Hospital Prognostic Score, Lung Immuno-oncology Prognostic Score 3, Lung Immuno-oncology Prognostic Score 4, score published by Holtzman et al., and Glasgow Prognostic Score) and outcomes in metastatic NSCLC treated in a first-line setting either with ICI in monotherapy (cohort 1; *n* = 75), ICI + CT (cohort 2; *n* = 56), or CT alone (cohort 3; *n* = 221). In the three cohorts, the biomarkers/scores were moderately associated with overall survival (OS) and progression-free survival (PFS). Their prognostic performance was relatively poor, with a maximum c-index of 0.66. None of them was specific to ICIs and could help to choose the best treatment modality. The systemic inflammation/nutritional status, associated with outcomes independently of the treatment, is therefore prognostic but not predictive in metastatic NSCLC.

## 1. Introduction

Immune checkpoint inhibitors (ICIs), in particular anti-programmed death 1 (PD-1)/programmed death 1 ligand 1 (PD-L1) antibodies, such as pembrolizumab, atezolizumab, or cemiplimab, are now widely used in the first-line treatment of metastatic/advanced-stage non-small-cell lung cancer (NSCLC) alone or in combination with platinum-doublet chemotherapy (CT). This is because it has been demonstrated that they significantly improved progression-free survival (PFS) and overall survival (OS) [1,2,3,4,5,6,7,8]. Unfortunately, the response to ICIs is variable. Indeed, while up to 15% of patients can benefit from long-term survival (rarely seen before the era of ICIs) [9,10,11,12], some do not respond or even suffer from a dramatic hyperprogression [13]. Therefore, identifying biomarkers that predict a response to ICIs is crucial.

To date, the only biomarker validated and used in clinical practice is the level of expression of PD-L1 in tumor cells, evaluated by immunohistochemistry on tumor specimens as a tumor proportion score (TPS) [14]. High PD-L1 expression, defined as a TPS ≥50%, is associated with a better response and long-term survival in patients treated with ICIs in monotherapy [12]. In the presence of a high PD-L1 expression, patients can be treated with ICIs in monotherapy or with ICIs in combination with a platinum-doublet CT (ICI + CT). A combination to CT is required if the PD-L1 expression is not high (TPS < 50%). It has also been reported that, compared to high PD-L1 TPS (≥50%), very high PD-L1 TPS (≥90%) was associated with even better outcomes in patients treated with ICIs in monotherapy, suggesting that the higher the PD-L1 expression, the better the response [15]. However, PD-L1 is not a perfect biomarker, given that not all tumors with high PD-L1 expression respond to ICIs, and that some tumors with a low or negative PD-L1 expression can respond to ICIs [14]. One reason explaining this imperfection could be the heterogeneous expression of PD-L1 within the tumors and through time and treatment lines [16,17]. Moreover, for tumors with a PD-L1 TPS ≥ 50%, there is currently no clear answer to whether the treatment should be ICIs in monotherapy or ICI + CT [18].

To address this imperfection of PD-L1, other experimental biomarkers have been studied, such as tumor mutation burden (TMB). A retrospective study recently reported that a high TMB was associated with better outcomes in patients treated with ICIs in monotherapy, independently of the PD-L1 expression (<1%, 1–49%, ≥50%) [19]. This could be explained by a microenvironment making the tumor more sensitive to ICIs in cases of high TMB levels, with an increased tumor infiltration by T cells positive for CD8 and PD-1, a higher proportion of tumor cells expressing PD-L1, and activation of the innate and adaptive immune response [19]. The authors also suggested that a very high TMB level could be used to select patients with PD-L1-positive NSCLC who would benefit from ICIs in monotherapy with no need to add CT [19]. Previous studies have also suggested that some genetic alterations in the tumor cells, such as somatic mutations in serine/threonine kinase 11 (*STK11*) or in kelch-like ECH-associated protein 1 (*KEAP1*), could serve as biomarkers to predict responses to ICIs in NSCLC [20,21]. However, a retrospective study including 2276 patients treated with ICIs, CT, epidermal growth factor receptor (*EGFR*) tyrosine kinase inhibitors (TKIs), or vascular endothelial growth factor (VEGF) inhibitors for an advanced-stage NSCLC later reported that somatic mutations in *STK11* or *KEAP1* were associated with shorter PFS and OS independently of the treatment. Therefore, they were prognostic factors but not predictive biomarkers for ICIs [22]. Other biomarkers have been proposed based on the analysis of T cells in the tumor microenvironment, but they are not used in routine clinical practice as they are expensive, time-consuming, tissue specimen-costly, and, most importantly, have limited reliability [14].

After systemic inflammation has been proposed as a hallmark of cancer based on the fact that it promotes tumor development and dissemination [23,24], various studies have investigated the role of biomarkers of systemic inflammation in response to ICIs in patients suffering from advanced-stage NSCLC [25]. Similarly, because the nutritional status can impact the response to treatment in various malignancies, biomarkers of the nutritional status have also been developed [26]. In contrast with more experimental biomarkers, these are derived from basic, routinely collected biological or clinical parameters that are widely available, such as hemogram, albumin, C-reactive protein (CRP), lactate dehydrogenase (LDH), or body mass index (BMI) [25,27,28]. Meanwhile, several scores, based on biomarkers of systemic inflammation and nutritional status, have also been developed and studied in patients with NSCLC treated with ICIs, such as the Lung Immune Prognostic Index (LIPI) [29], the Modified Lung Immune Prognostic Index (mLIPI) [30], the Scottish Inflammatory Prognostic Score (SIPS) [31], the EPSILoN [32], the Gustave Roussy Immune Score (GRIm) [33], the Royal Marsden Hospital Prognostic Score (RMH) [34], the Lung Immune Prognostic Score (LIPS) [35], the score developed by Holtzman et al. [36], and the Glasgow Prognostic Score (GPS) [37]. Many of these scores demonstrated a prognostic effect, as they were generally associated with outcomes. Nevertheless, it is still controversial whether they can predict the response to ICIs or whether they simply point out a group of patients with a bad or good prognosis, independently of the treatment’s nature, similar to what was demonstrated with somatic mutations in *STK11* or *KEAP1* [22]. Indeed, as most of the studies did not compare the association between the biomarkers/scores and the outcomes in cohorts of patients treated without ICIs, it is impossible to differentiate a predictive from a prognostic effect [30,31,32,34,35,37,38,39]. Furthermore, few studies have included patients treated with ICIs in the first-line setting, or they have mixed pretreated and treatment-naïve patients, whereas ICIs are currently mainly used in the first-line and PFS or OS are usually shorter in the second/third lines [27,29,32,40,41,42]. Due to its myelotoxic effect, recent prior CT could also have influenced the biomarkers’ assessment before ICIs.

To address these limitations, we conducted a study to compare the association between various scores reflecting the systemic inflammation/nutritional status and outcomes in patients with metastatic NSCLC treated in the first line either with an ICI in monotherapy, with an ICI in combination with CT, or with CT alone.

## 2. Results

### 2.1. Patients’ Characteristics

**Figure 1:**
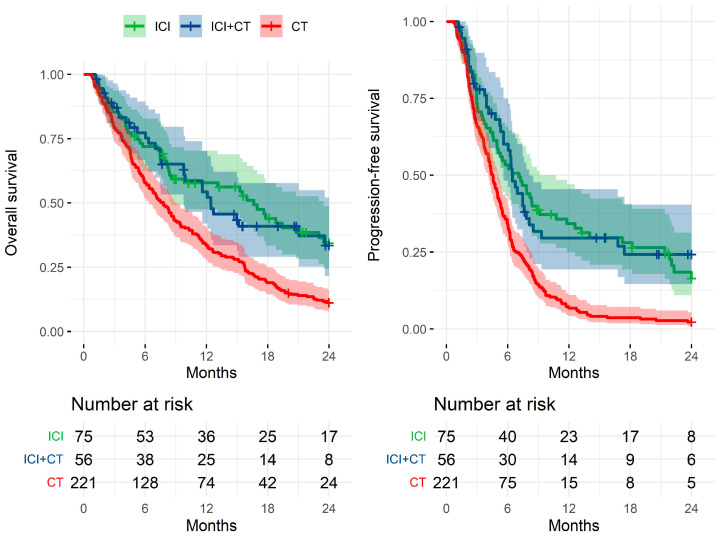
The Kaplan–Meier curves of overall survival and progression-free survival in the three cohorts of patients, treated with ICI + CT, ICI in monotherapy, or CT alone. Abbreviations: CT—chemotherapy, ICI—immune checkpoint inhibitor, and ICI + CT—ICI in combination with CT.

A total of 352 patients treated in our institution for metastatic NSCLC between 1 January 2008 and 1 October 2022 met the inclusion criteria. The first-line treatment consisted of ICI in monotherapy for 75 patients (cohort 1), ICI + CT for 56 patients (cohort 2), and CT alone for 221 patients (cohort 3). Table 1 displays the characteristics of patients in the three cohorts. The median age was 69 years in cohort 1, 65 years in cohort 2, and 63 years in cohort 3. There were 63% male patients in cohort 1, 55% in cohort 2, and 74% in cohort 3. Eastern Cooperative Oncology Group Performance Status (ECOG PS) was generally poorer (≥2) in cohort 1 (33%) than in cohorts 2 (23%) and 3 (23%). The histological subtype was squamous cell carcinoma in 28% of patients in cohort 1, 9% in cohort 2, and 24% in cohort 3. More patients had more than five metastases in cohort 2 (77% vs. 63% in cohort 1 and 57% in cohort 3). Brain metastases were more frequent in cohort 1 (35%) than in cohorts 2 (25%) and 3 (24%), while liver metastases were more common in cohort 3 (21%) than in cohorts 1 (11%) and 2 (18%). Figure 1 displays the Kaplan–Meier OS and PFS curves for the three cohorts. The median follow-up in surviving patients was 18 months in cohort 1, 16 months in cohort 2, and 68 months in cohort 3. As expected, the median PFS was longer in cohorts 1 and 2 (216 and 197 days, respectively) than in cohort 3 (138 days). The median OS had the same trend (506 and 378 days in cohorts 1 and 2, respectively, vs. 237 days in cohort 3).

### 2.2. Association between Scores and One-Year OS and Six-Month PFS

**Figure 2:**
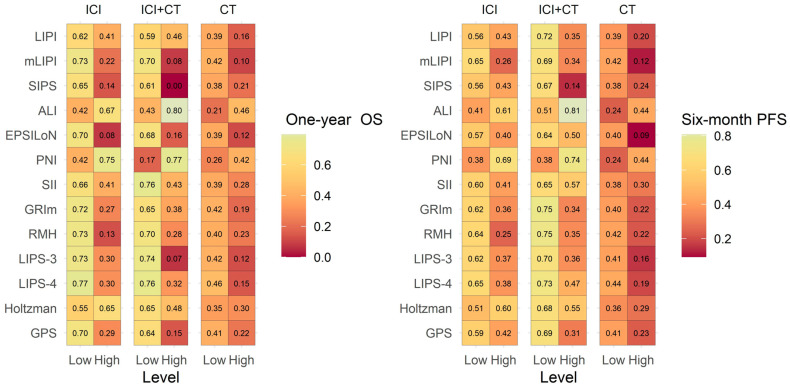
One-year overall survival (OS) and six-month progression-free survival (PFS) in the three cohorts, according to the scores based on biomarkers of systemic inflammation/nutritional status, analyzed as binary variables (high or low level). The numbers indicated in the squares represent the Kaplan–Meier estimation of the one-year OS or the six-month PFS. Abbreviations: ALI—Advanced Lung Cancer Inflammation Index [28], CT—chemotherapy alone, EPSILoN [32], GPS—Glasgow Prognostic Score [46], GRIm—Gustave Roussy Immune Score [33], Holtzman—score described by Holtzman et al. [36], ICI—ICI in monotherapy, ICI + CT—ICI in combination with chemotherapy, LIPI—Lung Immune Prognostic Index [29], LIPS-3—Lung Immuno-oncology Prognostic Score 3 [35], LIPS-4—Lung Immuno-oncology Prognostic Score 4 [35], mLIPI—Modified Lung Immune Prognostic Index [30], PNI—Prognostic Nutritional Index [43], RMH—Royal Marsden Hospital Prognostic Score [45], SII—Systemic Immune-Inflammation Index [44], and SIPS—Scottish Inflammatory Prognostic Score [31].

Scores based on biomarkers of systemic inflammation/nutritional status were assessed in the three cohorts (Table 2, Supplementary Table S1, and Supplementary Figure S1). Figure 2 represents the one-year OS and six-month PFS for the high and low score values in the three cohorts. For a given score and treatment type, the greater the difference in one-year OS/six-month PFS between low and high values, the more it was associated with one-year OS/six-month PFS. As illustrated with colors, favorable score values were generally associated with better OS/PFS in the three cohorts, meaning that a lower systemic inflammation or better nutritional status at baseline was associated with better survival, independent of treatment. Scores were thus prognostic in the three cohorts. However, as opposed to the others, the prognostic value of the score developed by Holtzman et al. [36] appeared poorer in the cohort of patients treated with ICI in monotherapy, as the one-year OS/six-month PFS of patients with high values at baseline (thus with an unfavorable status at baseline) were surprisingly better than the one-year OS/six-month PFS of patients with low values at baseline (thus with a presumed favorable status at baseline). Please note that, in contrast to the other scores, for the Advanced Lung Cancer Inflammation Index (ALI) and the Prognostic Nutritional Index (PNI), higher values were associated with lower systemic inflammation and better nutritional status, respectively. It is therefore not surprising that high values for ALI and PNI were associated with a better prognosis.

### 2.3. Prognostic Value of Scores

We estimated the prognostic performance of the scores based on biomarkers of systemic inflammation/nutritional status using the concordance index (c-index). This index ranges from 0 to 1 and can be interpreted as the proportion of patient pairs for which there is an agreement between predicted and observed survival times, i.e., the patient with the longest predicted survival time also has the longest observed survival time [47]. A c-Index of 0.5 thus represents the absence of concordance between the score and the survival time, while a value of 1.0 represents perfect concordance. The c-index of each score was calculated twice: (1) with dichotomized scores (treated as a binary variable, with high and low values corresponding to a score ≥ median or < median, respectively), and (2) with non-dichotomized scores (using a non-linear relationship between the score and survival). Their prognostic performance was generally poor (Table 3), as the highest c-index value was only at 0.66, meaning that more favorable score values were associated with better survival in only 66% of the pairs of patients. The score developed by Holtzman et al. [36] had the poorest prognostic performance with a c-index value below 0.5, meaning that the more favorable score values were more associated with shorter survival.

Sensitivity analysis performed with dichotomized scores on patients with no missing score values, on patients with non-squamous NSCLC, and on balanced groups (in terms of sample size) showed similar results (Supplementary Table S2).

### 2.4. Interaction between Scores and Cohort Differences

To assess if a score had a greater prognostic value in one cohort versus another and, therefore, a predictive value for one particular treatment, we calculated the hazard ratios (HRs) of the interaction between the cohorts and the scores based on biomarkers of systemic inflammation/nutritional status. These HRs compare the cohort differences (1 vs. 2 or 2 vs. 3) when the score is high to the cohort differences when the score is low. An HR of 1 indicates an absence of interaction, i.e., that the cohort differences in patients with high scores are identical to those in patients with low scores. As the most common decision encountered today is whether to use ICI in monotherapy or ICI + CT, the most important HRs are those of the interaction for the differences between cohort 1 (ICI in monotherapy) and cohort 2 (ICI + CT) for OS, as reported in the column “Interaction ICI + CT vs. ICI, OS” of Table 4. In this column, HRs can be interpreted as follows: HR = 1 means that the effects of the score on OS are the same in cohorts 1 (ICI in monotherapy) and 2 (ICI + CT); HR = 2 means that a high score increases the risk of death twice more in cohort 1 (ICI in monotherapy) than in cohort 2 (ICI + CT); and HR = 0.5 means that a high score increases the risk of death twice more in cohort 2 (ICI + CT) than in cohort 1 (ICI in monotherapy).

The interaction measured for the SIPS score [31] was the only one that was statistically significant (HR = 0.24; 0.08–0.75) for this column, with an elevated SIPS score at baseline increasing four times the risk of death in cohort 2 (ICI + CT) as compared to cohort 1 (ICI in monotherapy). More interactions were observed when comparing cohort 2 (ICI + CT) to cohort 3 (CT).

The sensitivity analysis performed for the HRs of the interactions for the difference between cohorts 1 and 2 on patients with no missing score values and on balanced groups (in terms of sample size) showed similar results (Supplementary Table S3). Lower HRs for interaction between cohort 1 (ICI in monotherapy) and cohort 2 (ICI + CT) were observed for some scores (i.e., mLIPI, LIPS-3, GPS) when analyzing non-squamous NSCLCs only (Supplementary Table S3), indicating that high scores might increase the risk of death more in cohort 2 (ICI + CT) than in cohort 1 (ICI in monotherapy) in the case of non-squamous NSCLC.

## 3. Discussion

Our study reports the association of various scores reflecting the systemic inflammation/nutritional status with OS and PFS in three cohorts of patients treated for a metastatic NSCLC, either with ICI in monotherapy, ICI + CT, or CT alone in the first-line setting. In the three cohorts, the scores were associated with the risk of death or progression. However, their prognostic performance was poor, as the highest c-index was calculated at 0.66, which is close to 0.50, i.e., the absence of concordance between the score values and the survival. The score developed by Holtzman et al. [36] had the poorest prognostic performance, with a c-index even below 0.50. For patients with higher scores at baseline, the one-year OS was also curiously higher for patients treated with ICI in monotherapy (65%) than with ICI + CT (48%), whereas it was the opposite in the study that established this score [36]. The scores were also not specifically predictive of the response to a particular treatment modality, except maybe for the SIPS score, for which higher scores were associated with a greater risk of death in the cohort treated with ICI + CT than in the one treated with ICI in monotherapy. This score was created and studied in a cohort of patients treated with ICI in monotherapy and not with ICI + CT [31]. Moreover, the interaction observed in our study has to be considered with caution, given the small sample size and the high number of assessed scores. In our study, the interaction was also observed for some scores (i.e., mLIPI, LIPS-3, GPS) when analyzing patients with only non-squamous NSCLC, indicating that high scores could increase the risk of death in those treated with ICI + CT compared to those treated with ICI in monotherapy in this histological subtype. However, it should be interpreted with the utmost caution, as the sample size was very limited after excluding squamous NSCLC and the magnitude of the interaction was low.

It is now well established that systemic inflammation plays a critical role in many phases of cancer development, influences responses to therapies, and is involved in cancer cachexia, which is itself a poor prognostic factor [24,48]. It is therefore not surprising to observe that the systemic inflammation/nutritional status is prognostic, independent of the treatment. This is consistent with previous studies conducted before the era of immunotherapy, already showing an association between biomarkers of systemic inflammation/nutritional status (PNI, neutrophil-to-lymphocyte ratio (NLR), Systemic Immune-Inflammation Index (SII)) and outcomes in patients treated for an NSCLC [49,50,51,52]. The same link has also been described in extra-thoracic malignancies, such as diffuse large B-cell lymphoma, melanoma, colorectal, head and neck, liver, prostate, or renal cancers [53,54].

Furthermore, multiple studies have tried to find scores that would specifically predict response to ICIs. One of them reported that the LIPI was associated with OS and PFS in a cohort of 431 patients treated with ICI in monotherapy but not in a cohort of 157 patients treated with CT only, hypothesizing that the LIPI would be predictive of response to ICIs [29]. Nevertheless, their results were contested later by another study that performed a secondary analysis of pooled data from four trials. They reported an association between the LIPI and the outcomes not only in 1489 patients treated with atezolizumab but also in 687 patients treated with docetaxel, concluding that this score was simply prognostic [40]. Another pooled analysis of 5 randomized trials evaluating ICIs versus CT only and 6 randomized trials evaluating targeted therapies (TTs) versus other TTs or CT only concluded also that the LIPI was prognostic in all the cohorts (1368 patients treated with ICIs and 1072 patients treated with CT only for the trials evaluating ICIs; 1110 patients treated with TTs and 437 patients treated with CT only for the trials evaluating TTs) [41]. A meta-analysis published a few years later finally concluded that the LIPI was prognostic, independent of the treatment [55].

However, it should be highlighted that the aforementioned studies over the LIPI evaluated the score in cohorts of patients treated with different therapeutic modalities. In contrast, many of the studies that established an association between systemic inflammation/nutritional status and outcomes in patients treated with ICIs, such as those that evaluated the Scottish Inflammatory Prognostic Score (SIPS) [31], EPSILoN [32,56,57], the Prognostic Nutritional Index (PNI) [58,59,60,61], the SII [39], the Gustave Roussy Immune score (GRIm) [33,34], the Royal Marsden Hospital Prognostic Score (RMH) [34], the Lung Immuno-oncology Prognostic Score 3 (LIPS-3), the Lung Immuno-oncology Prognostic Score 4 (LIPS-4) [35], and the Glasgow Prognostic Score (GPS) [37] scores, did not test their results in cohorts of patients treated with other modalities. This prevented them from concluding that their biomarkers/scores were specific for ICIs.

More importantly, few studies evaluating the systemic inflammation/nutritional status addressed the issue of the treatment choice in patients suffering from an NSCLC with PD-L1 TPS ≥50%, as these can be treated either with ICI in monotherapy or with ICI + CT [18]. Currently, in these cases, physicians rely on clinical and/or pathological/molecular characteristics to choose between the two treatment modalities [62]. Recently, a study reported that, in contrast to ICI in monotherapy, the ALI score was less associated with outcomes in a cohort of 444 patients treated with CT alone and lost its predictive power in a cohort of 212 patients treated with ICI + CT, raising the question of whether it would be ICI-specific [27]. In NSCLC with PD-L1 TPS ≥50%, a high ALI score (>18) was also associated with longer OS in patients treated with ICI in monotherapy (*n* = 156) but not in those treated with ICI + CT (*n* = 38) [27]. As opposed to these results, our study did not detect any score that could guide the treatment choice for these patients. That seems logical given the general prognostic nature of the systemic inflammation/nutritional status.

To our best knowledge, our study is the first to evaluate the association of a large panel of biomarkers/scores reflecting the systemic inflammation/nutritional status with the response to first-line treatment in metastatic NSCLC. Nevertheless, several limitations should be pointed out, mainly due to the study’s retrospective nature. There were missing data, but there were not many, given that the biomarkers/scores were based on very common clinical/biological data. As a single-center study, the number of patients was also limited, particularly in cohort 2. Moreover, only 35% of the NSCLCs in cohorts 1 and 2 had a PD-L1 TPS <50%, which could be disturbing given that this proportion is generally estimated between 72 and 77% in the literature [63,64]. This is explained by the fact that the patients were included consecutively and that ICI + CT was implemented later than ICI in monotherapy. The recent introduction of ICIs in the treatment of NSCLC also explains why our work does not concern long-term survival, the follow-up time in cohorts 1 and 2 not being long enough to determine the three-year or five-year PFS/OS. The characteristics of the three cohorts were also not identical, but the differences were not important and reflected real-life practice. Moreover, 100% of the patients were of Caucasian origin, which can be explained by the fact that our institution is located in the countryside, far from the big Belgian cities where most non-Caucasians live. Therefore, we cannot extend our conclusions to patients of other ethnic origins. Finally, while some studies reported interest in evaluating biomarkers/scores after treatment initiation [65,66], we decided to focus only on pre-treatment values as it is at baseline that physicians have to choose the first-line therapeutic modality.

## 4. Materials and Methods

### 4.1. Study Design and Population

This is a single-center, retrospective study conducted in a Belgian academic hospital. All patients treated for a metastatic NSCLC between 1 January 2008 and 1 October 2022 were included. Patients who experienced cancer recurrence after a presumed curative surgery with or without adjuvant CT or after concurrent chemoradiation without consolidative ICI were not excluded. Patients with an NSCLC harboring an EGFR gene mutation or an anaplastic lymphoma kinase (ALK) gene rearrangement or treated with a first-line tyrosine kinase inhibitor were excluded.

We divided the study population into three cohorts, according to the first-line therapeutic modality: cohort 1 for patients treated with ICI in monotherapy; cohort 2 for those treated with ICI + CT; and cohort 3 for those treated with platinum-doublet CT alone, without ICI in further lines. 

### 4.2. Data Collection and Definitions

Clinical, biological, radiological, and pathological data were extracted from medical records and anonymized before analysis. Thirteen scores reflecting the systemic inflammation/nutritional status based on previous publications were assessed at baseline within 30 days of the first treatment cycle (Table 2). PD-L1 expression was evaluated by immunohistochemistry on a tumor specimen and divided into three levels of expression: <1%, 1–49%, and ≥50%. Patients were treated according to the latest guidelines available and were evaluated every two or three cycles by radiological assessment.

### 4.3. Statistical Methods

Baseline characteristics are presented as medians (P25; P75) for continuous variables and as numbers and proportions for categorical variables. Continuous scores (ALI, PNI, and SII) were log-transformed in order to obtain a more symmetric distribution (Supplementary Figure S1). According to the STROBE statement, baseline characteristics are described without inferential measures [67]. Due to the retrospective nature of this study, some biomarker values could not be calculated. This concerned 1.9%, 0.0%, and 10.5% of cohorts 1, 2, and 3, respectively. Missing scores were imputed using the MICE algorithm [68]. Except otherwise stated, scores were analyzed as binary variables with high and low values corresponding to a score ≥median or <median, respectively. This allowed an easier comparison of different scores.

Survival curves were generated using the Kaplan–Meier method [69]. OS was calculated from the date of the first treatment cycle until death from any cause or the date of the data cut-off (1 October 2022). PFS was calculated from the date of the first treatment cycle until radiological progression or death from any cause or the date of the data cut-off. The median follow-up was estimated in surviving patients. In order to describe the association between each score and the survival in the three cohorts, the one-year OS and the six-month PFS were calculated in each of the six subgroups (three cohorts × two score levels).

The concordance index (c-index) was used to assess the scores’ prognostic performances. In order to respect the discontinuous nature of the majority of the scores and the way their values were used to categorize patients, the c-index was computed twice for each score: (1) scores dichotomized (treated as a binary variable), and (2) scores non-dichotomized using a non-linear relationship between the score and survival, i.e., 1 prediction by score category if the score has ≤6 possible values or a restricted cubic spline with 5 knots if the score has >6 possible values (this approach concerned the following scores: ALI, SII, and PNI.

The interaction between cohorts and scores was analyzed through Cox proportional hazard regression models [70]. Hazard ratios (HRs) of the interactions were computed for the difference between cohorts 1 and 2 and for the difference between cohorts 2 and 3.

A sensitivity analysis was performed for four outcomes (c-index for OS and PFS using dichotomized scores and HRs of the interactions for the difference between cohorts 1 and 2 for OS and PFS) on four populations: (1) the whole population, including patients with missing scores imputed by the MICE algorithm [68] (main analysis); (2) the population with complete data only, removing patients with missing data; (3) the population with the three cohorts balanced in sample size (the c-index and HR were the median values of 200 analyses on balanced data); and (4) patients with non-squamous NSCLC.

The statistical analyses were performed using R 4.1.1 and the following packages: survival and survminer (for the analysis of survival curves), MICE (for the completion of missing scores), rms (for the c-Index and the restricted cubic splines), and ggplot2 (for graphical representations) [71].

### 4.4. Ethical Approval

This study was conducted in accordance with the Declaration of Helsinki and approved by the institutional ethics committee (Comité d’éthique hospitalo-facultaire) of CHU UCL Namur (Godinne Site), Belgium (internal protocol code 144/2022, 8 November 2022). Since it is a retrospective study, informed consent of the participants was waived by the ethics committee. 

## 5. Conclusions

Our study demonstrates that biomarkers/scores reflecting the systemic inflammation/nutritional status are moderately associated with outcomes in metastatic NSCLC, independent of the treatment modality. Therefore, they are currently useless to guide the treatment choice between ICI in monotherapy or ICI + CT. We hope that the results of prospective randomized trials, such as the PERSEE trial evaluating pembrolizumab versus pembrolizumab in combination with CT in advanced-stage NSCLC with PD-L1 TPS ≥50%, will provide some answers [72]. Investigators of studies evaluating biomarkers predicting the response to ICIs should also remember to evaluate patients treated with other treatment modalities in order to distinguish between a predictive (i.e., specific to ICIs) and a prognostic effect.

## Figures and Tables

**Table 1:** Patients’ characteristics in the three cohorts.

	Cohort 1 (*n* = 75) ICI Median (P25; P75) or *n* (%)	Cohort 2 (*n* = 56) ICI + CT Median (P25; P75) or *n* (%)	Cohort 3 (*n* = 221) CT Median (P25; P75) or *n* (%)
**Clinical characteristics**			
Age, years	69 (62; 74)	65 (57; 70.25)	63 (56; 68)
Sex, male	47 (63)	31 (55)	164 (74)
Smoking status			
Current	39 (52)	28 (50)	124 (56)
Former	34 (45)	26 (46)	87 (39)
Never	2 (3)	2 (4)	10 (5)
ECOG PS at diagnosis			
0	19 (25)	12 (21)	47 (21)
1	31 (41)	31 (55)	123 (56)
2	19 (25)	11 (20)	46 (21)
3	6 (8)	2 (4)	3 (1)
4	0 (0)	0 (0)	2 (1)
BMI, kg/m²	24 (21; 27)	25 (22; 27)	24 (21; 28)
**Pathological characteristics**			
NSCLC histological subtype Squamous	21 (28)	5 (9)	53 (24)
Non-squamous	54 (72)	51 (91)	168 (76)
PD-L1 TPS			
<1%	0 (0)	25 (45)	15 (7)
1–49%	0 (0)	21 (37)	6 (3)
≥50%	75 (100)	8 (14)	4 (2)
Missing	0 (0)	2 (4)	196 (89)
**Relapse after**			
Surgery	10 (13)	8 (14)	13 (6)
With adjuvant CT	2 (3)	2 (4)	7 (3)
SRT	2 (3)	0 (0.0)	4 (2)
Concurrent CRT	2 (3)	3 (5)	10 (5)
**Metastases**, number			
1	14 (19)	8 (14)	54 (24)
2–5	14 (19)	5 (9)	42 (19)
>5	47 (63)	43 (77)	125 (57)
**Metastases**, localization			
Brain	26 (35)	14 (25)	54 (24)
Liver	8 (11)	10 (18)	46 (21)
**Treatment**			
Pembrolizumab	71 (95)	-	-
Atezolizumab	4 (5)	-	-
Cis/pem/pembro	-	35 (62)	-
Carbo/pem/pembro	-	16 (29)	-
Carbo/pacli/pembro	-	5 (9)	-
Cis/pem	-	-	59 (27)
Carbo/pem	-	-	3 (1)
Carbo/pacli	-	-	1 (0)
Cis/gemci	-	-	37 (17)
Carbo/gemci	-	-	18 (8)
Cis/vino	-	-	66 (30)
Carbo/vino	-	-	29 (13)
Cis/eto	-	-	6 (3)
Cis/doce	-	-	2 (1)
**Outcome**			
PFS, days	216 (90; 651)	197 (119; 530)	138 (76; 208)
OS, days	506 (160;1015)	378 (193; NR)	237 (114; 479)

Abbreviations: BMI—body mass index, carbo—carboplatin, Cis—cisplatin, CRT—chemoradiation, CT—chemotherapy, doce—docetaxel, ECOG PS—Eastern Cooperative Oncology Group Performance Status, eto—etoposide, gemci—gemcitabine, ICI—immune checkpoint inhibitor, ICI + CT—ICI in combination with chemotherapy, n—number, NR—not reached, NSCLC—non-small-cell lung cancer, P—percentile, OS—overall survival, pacli—paclitaxel, pembro—pembrolizumab, pem—pemetrexed, PFS—progression-free survival, SRT—stereotactic radiotherapy, TPS—tumor proportion score, and vino—vinorelbine.

**Table 2:** Scores based on biomarkers of systemic inflammation/nutritional status evaluated in the study.

Scores	Biomarkers	Assessment
Lung Immune Prognostic Index (LIPI) [29]	dNLR LDH	dNLR > 3: 1 point LDH > ULN: 1 point Total points: 0: good; 1: intermediate; 2: poor
Modified Lung Immune Prognostic Index (mLIPI) [30]	ECOG PS LDH NLR	ECOG PS = 1 or 2: 1 point NLR > 3: 1 point LDH > 1.5 × ULN: 1 point Total points: 0: good; 1: intermediate; 2: poor; 3: very poor
Scottish Inflammatory Prognostic Score (SIPS) [31]	Albumin Neutrophil	Albumin < 35 g/L: 1 point Neutrophil count > 7.5 × 10^9^/L: 1 point Total points: 0: good; 1: intermediate; 2: poor
Advanced Lung Cancer Inflammation Index (ALI) [28]	BMI Albumin NLR	$\frac{\mathrm{BMI}\;\left(\frac{\mathrm{kg}}{m^{2}}\right)\;\times \;\mathrm{albumin}\;\left(\frac{g}{\mathrm{dL}}\right)}{\mathrm{NLR}}$ Low: high systemic inflammation; High: low systemic inflammation
EPSILoN [32]	ECOG PS Smoking Liver metastases LDH NLR	ECOG PS ≥ 2: 1 point Smoking < 43 pack-years: 1 point Liver metastases: 1 point LDH > 400 mg/dL: 1 point NLR > 4: 1 point Total points: 0: good; 1–2: intermediate; 3–5: poor
Prognostic Nutritional Index (PNI) [43]	Albumin Lymphocyte	10 × Albumin (g/dL) + 0.005 × Lymphocyte count /mm^3^ Low: poor nutritional status; high: good nutritional status
Systemic Immune-Inflammation Index (SII) [44]	Platelet Neutrophil Lymphocyte	Platelet count × Neutrophil count/Lymphocyte count Low: low systemic inflammation; High: high systemic inflammation
Gustave Roussy Immune Score (GRIm) [33]	LDH Albumin NLR	LDH > ULN: 1 point Albumin < 35 g/L: 1 point NLR > 6: 1 point Total points: 0–1: low risk; 2–3: high risk
Royal Marsden Hospital Prognostic Score (RMH) [45]	LDH Albumin Site of metastasis	LDH > ULN: 1 point Albumin > 35 g/L: 1 point Site of metastasis > 2: 1 point Total points: 0–1: low risk; 2–3: high risk
Lung Immuno-oncology Prognostic Score 3 (LIPS-3) [35]	ECOG PS Pretreatment steroids NLR	ECOG PS ≥ 2: 1 point Pretreatment steroids: 1 point NLR ≥ 4: 1 point Total points: 0: favorable; 1–2: intermediate; 3: poor
Lung Immuno-oncology Prognostic Score 4 (LIPS-4) [35]	ECOG PS Pretreatment steroids NLR LDH	ECOG PS ≥ 2: 1 point Pretreatment steroids: 1 point NLR ≥ 4: 1 point LDH ≥ 252 U/L: 1 point Total points: 0: favorable; 1–2: intermediate; 3–4: poor
Holtzman Score [36]	Age Sex Smoking Histology dNLR	Age ≥ 65 years: 1 point Female sex: 1 point Never-smoker: 1 point Adenocarcinoma: 1 point dNLR ≥ 3: 1 point Total points: 0–2: favorable; 3–5: poor
Glasgow Prognostic Score (GPS) [46]	CRP Albumin	CRP > 10 mg/L: 1 point Albumin < 35 g/L: 1 point Total points: 0: good; 1: intermediate; 2: poor

Abbreviations: BMI—body mass index, CRP—C-reactive protein, dNLR—derived neutrophil to lymphocyte ratio = neutrophil count/(white blood cell count—neutrophil count), ECOG PS—Eastern Cooperative Oncology Group Performance Status, LDH—lactate dehydrogenase, NLR—neutrophil to lymphocyte ratio = neutrophil count/lymphocyte count, and ULN—upper limit of normal.

**Table 3:** Concordance index (c-index) of the scores based on biomarkers of systemic inflammation/nutritional status.

Score	OS-D	OS-ND	PFS-D	PFS-ND
LIPI	0.58	0.62	0.57	0.60
mLIPI	0.63	0.66	0.60	0.61
SIPS	0.59	0.64	0.57	0.61
ALI	0.60	0.63	0.57	0.60
EPSILoN	0.59	0.63	0.57	0.60
PNI	0.60	0.63	0.59	0.61
SII	0.57	0.61	0.54	0.58
GRIm	0.62	0.66	0.59	0.63
RMH	0.63	0.66	0.60	0.64
LIPS-3	0.62	0.64	0.59	0.60
LIPS-4	0.63	0.66	0.60	0.62
Holtzman	0.49	0.53	0.50	0.52
GPS	0.61	0.62	0.58	0.59

Abbreviations: ALI—Advanced Lung Cancer Inflammation Index [28], D—dichotomized scores, EPSILoN [32], GPS—Glasgow Prognostic Score [46], GRIm—Gustave Roussy Immune Score [33], Holtzman—score described by Holtzman et al. [36], LIPI—Lung Immune Prognostic Index [29], LIPS-3—Lung Immuno-oncology Prognostic Score 3 [35], LIPS-4—Lung Immuno-oncology Prognostic Score 4 [35], mLIPI—Modified Lung Immune Prognostic Index [30], ND—non-dichotomized scores, OS—overall survival, PFS—progression-free survival, PNI—Prognostic Nutritional Index [43], RMH—Royal Marsden Hospital Prognostic Score [45], SII—Systemic Immune-Inflammation Index [44], and SIPS—Scottish Inflammatory Prognostic Score [31].

**Table 4:** Interaction between scores based on biomarkers of systemic inflammation/nutritional status and cohort differences.

Scores	Interaction ICI + CT vs. ICI, OS		Interaction ICI + CT vs. ICI, PFS		Interaction ICI + CT vs. CT, OS		Interaction ICI + CT vs. CT, PFS	
	HR (95% CI)	*p*-Value	HR (95% CI)	*p*-Value	HR (95% CI)	*p*-Value	HR (95% CI)	*p*-Value
LIPI	0.98 (0.35–2.74)	0.97	0.72 (0.29–1.79)	0.47	1.24 (0.56–2.73)	0.60	0.93 (0.45–1.94)	0.85
mLIPI	0.61 (0.24–1.56)	0.30	0.60 (0.25–1.42)	0.24	0.44 (0.20–0.95)	0.037	0.54 (0.26–1.12)	0.099
SIPS	0.24 (0.08–0.75)	0.014	0.20 (0.07–0.60)	0.004	0.13 (0.05–0.34)	0.000036	0.13 (0.05–0.34)	0.000028
ALI	1.35 (0.46–3.96)	0.58	1.26 (0.51–3.15)	0.62	1.40 (0.55–3.55)	0.48	1.26 (0.57–2.80)	0.56
EPSILoN	1.31 (0.49–3.48)	0.59	1.02 (0.41–2.53)	0.97	0.77 (0.34–1.73)	0.52	1.12 (0.52–2.41)	0.77
PNI	1.21 (0.47–3.14)	0.69	1.07 (0.47–2.44)	0.87	2.70 (1.26–5.78)	0.011	1.96 (0.98–3.91)	0.058
SII	0.74 (0.26–2.10)	0.57	1.22 (0.51–2.90)	0.66	0.55 (0.23–1.33)	0.18	0.93 (0.45–1.94)	0.85
GRIm	1.03 (0.41–2.59)	0.95	0.94 (0.41–2.17)	0.89	0.68 (0.32–1.45)	0.32	0.70 (0.35–1.42)	0.33
RMH	1.52 (0.60–3.85)	0.38	1.39 (0.60–3.20)	0.45	0.64 (0.30–1.37)	0.25	0.54 (0.27–1.08)	0.082
LIPS-3	0.46 (0.18–1.17)	0.10	0.42 (0.18–0.98)	0.044	0.40 (0.18–0.85)	0.018	0.44 (0.22–0.91)	0.026
LIPS-4	1.02 (0.40–2.64)	0.96	0.81 (0.35–1.84)	0.61	0.77 (0.35–1.70)	0.52	0.69 (0.34–1.39)	0.30
Holtzman	0.60 (0.22–1.67)	0.33	0.55 (0.22–1.34)	0.19	0.85 (0.38–1.89)	0.69	0.78 (0.37–1.62)	0.50
GPS	0.50 (0.19–1.32)	0.16	0.62 (0.25–1.56)	0.31	0.28 (0.12–0.65)	0.0028	0.43 (0.19–0.96)	0.04

Abbreviations: ALI—Advanced Lung Cancer Inflammation Index [28], CI—confidence interval, CT—cohort of patients treated with CT in monotherapy, EPSILoN [32], GPS—Glasgow Prognostic Score [46], GRIm—Gustave Roussy Immune Score [33], Holtzman—score described by Holtzman et al. [36], HR—hazard ratio, ICI—cohort of patients treated with ICI in monotherapy, ICI + CT—cohort of patients treated with ICI in combination with chemotherapy, LIPI—Lung Immune Prognostic Index [29], LIPS-3—Lung Immuno-oncology Prognostic Score 3 [35], LIPS-4—Lung Immuno-oncology Prognostic Score 4 [35], mLIPI—Modified Lung Immune Prognostic Index [30], OS—overall survival, PFS—progression-free survival, PNI—Prognostic Nutritional Index [43], RMH—Royal Marsden Hospital Prognostic Score [45], SII—Systemic Immune-Inflammation Index [44], SIPS—Scottish Inflammatory Prognostic Score [31], and vs.—versus.

## Data Availability

The data presented in this study are available upon request from the corresponding author. They are not publicly available to ensure the strict confidentiality of the patients’ data.

## Supplementary Materials

The following supporting information can be downloaded at: https://www.mdpi.com/article/10.3390/ijms24043618/s1.
