# Supplementary material for: Systemic Inflammation/Nutritional Status Scores Are Prognostic but Not Predictive in Metastatic Non-Small-Cell Lung Cancer Treated with First-Line Immune Checkpoint Inhibitors
Source: Int J Mol Sci. 2023 Feb 10;24(4):3618. doi: 10.3390/ijms24043618 (PMC9966997; doi:10.3390/ijms24043618)
Supplement: Supplementary file 1 [file ijms-24-03618-s001.zip › ijms-2147741-supplementary.pdf]

Supplementary data

Table S1. Scores based on systemic inflammation/nutritional status in the three cohorts of patients, treated with ICI in monotherapy, ICI+CT, or CT alone.
Continuous scores (ALI, PNI, and SII) were log-transformed in order to obtain a more symmetric distribution.

**Scores**	**Cohort 1 (*n*=75) ICI Median (P25; P75) or *n* (%)**	**Cohort 2 (*n*=56) ICI+CT Median (P25; P75) or *n* (%)**	**Cohort 3 (*n*=221) CT Median (P25; P75) or *n* (%)**
**LIPI**			
0	35 (47)	9 (16)	70 (32)
1	25 (33)	29 (52)	95 (43)
2	14 (19)	18 (32)	54 (24)
Missing	1 (1)	0 (0)	2 (1)
**mLIPI**			
0	12 (16)	5 (9)	36 (16)
1	39 (52)	37 (66)	125 (57)
2	20 (27)	12 (21)	46 (21)
3	3 (4)	2 (4)	12 (5)
Missing	1 (1)	0 (0)	2 (1)
**SIPS**			
0	34 (45)	20 (36)	36 (16)
1	25 (33)	29 (52)	78 (35)
2	14 (19)	7 (12)	59 (27)
Missing	2 (3)	0 (0)	48 (22)
**ALI**			
Median (P25; P75)	1.25 (0.99; 1.38)	0.97 (0.80; 1.30)	1.09 (0.77; 1.38)
Missing	2	0	48
**EPSILoN**			
0	9 (12)	3 (5)	22 (10)
1-2	50 (67)	38 (68)	150 (68)
3-5	15 (20)	15 (27)	41 (19)
Missing	1 (1)	0 (0)	8 (4)
**PNI**			
Median (P25; P75)	1.63 (1.60; 1.67)	1.66 (1.60; 1.68)	1.62 (1.57; 1.68)
Missing	2	0	48
**SII**			
Median (P25; P75)	3.16 (2.92; 3.49)	3.53 (3.24; 3.73)	3.29 (2.99; 3.59)
Missing	0	0	0
**GRIm**			
0	25 (33)	12 (21)	29 (13)
1	22 (29)	23 (41)	72 (33)
2	21 (28)	15 (27)	36 (16)
3	4 (5)	6 (11)	36 (16)
Missing	3 (4)	0 (0)	48 (22)
**RMH**			
0	27 (36)	21 (38)	37 (17)
1	25 (33)	14 (25)	58 (26)
2	15 (20)	16 (29)	56 (25)
3	5 (7)	5 (9)	22 (10)
Missing	3 (4)	0 (0)	48 (22)
**LIPS-3**			
0	20 (27)	8 (14)	53 (24)
1	28 (37)	32 (57)	104 (47)

2	22 (29)	13 (23)	43 (19)
3	5 (7)	3 (5)	21 (10)
Missing	0 (0)	0 (0)	0 (0)
**LIPS-4**			
0	20 (27)	10 (18)	39 (18)
1	22 (29)	18 (32)	92 (42)
2	15 (20)	19 (34)	46 (21)
3	14 (19)	8 (14)	34 (15)
4	3 (4)	1 (2)	8 (4)
Missing	1 (1)	0 (0)	2 (1)
**Holtzman**			
0	3 (4)	1 (2)	11 (5)
1	15 (20)	8 (14)	44 (20)
2	37 (49)	14 (25)	102 (46)
3	13 (17)	23 (41)	54 (25)
4	7 (9)	10 (18)	9 (4)
Missing	0 (0)	0 (0)	1 (0)
**GPS**			
0	13 (17)	18 (32)	23 (10)
1	35 (47)	26 (46)	72 (33)
2	24 (32)	12 (21)	79 (36)
Missing	3 (4)	0 (0)	47 (21)

Abbreviations: ALI — Advanced Lung Cancer Inflammation Index [1], CT — chemotherapy alone, EPSILoN [2], GPS — Glasgow Prognostic Score [3], GRIm — Gustave Roussy Immune Score [4], Holtzman — score described by Holtzman et. al [5], ICI — ICI in monotherapy, ICI+CT — ICI in combination with chemotherapy, LIPI — Lung Immune Prognostic Index [6], LIPS-3 — Lung Immuno-oncology Prognostic Score 3 [7], LIPS-4 — Lung Immuno-oncology Prognostic Score 4 [7], mLIPI — Modified Lung Immune Prognostic Index [8], n — number, P — percentile, PNI — Prognostic Nutritional Index [9], RMH — Royal Marsden Hospital Prognostic Score [10], SII — Systemic Immune-Inflammation Index, expressed in 10^9 cells/L [11], and SIPS — Scottish Inflammatory Prognostic Score [12].

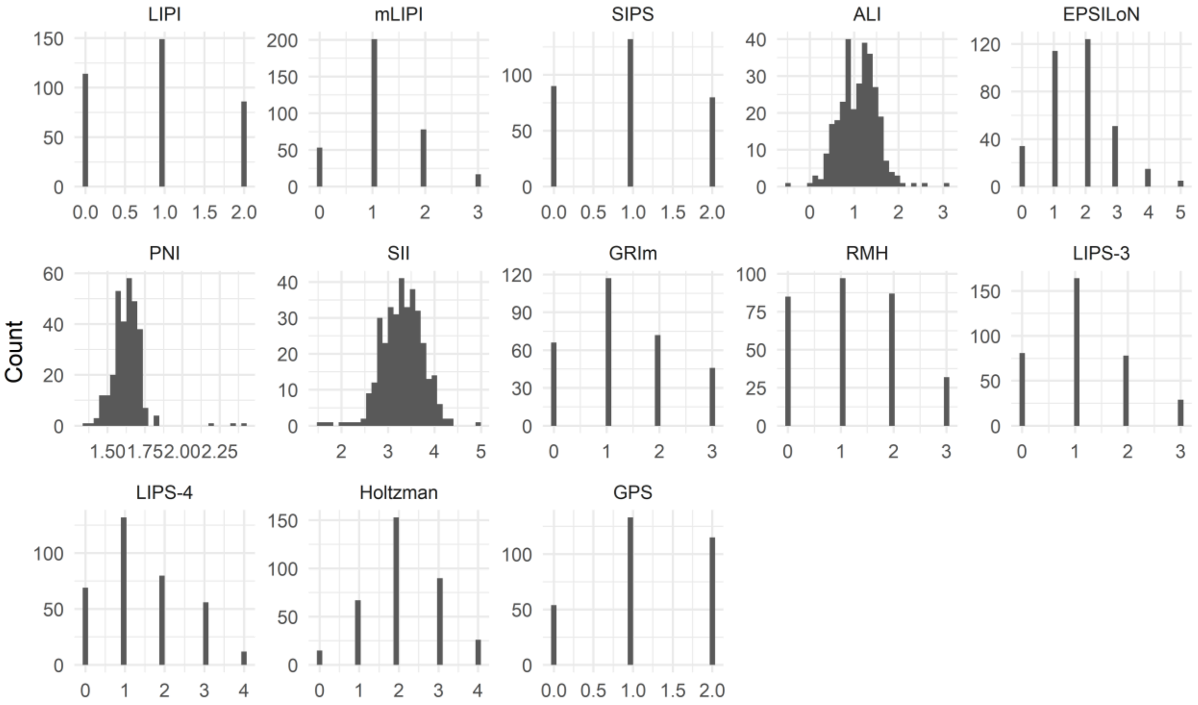

Figure S1. Distribution of the scores based on systemic inflammation/nutritional status. Continuous scores (ALI, PNI, and SII) were log-transformed in order to obtain a more symmetric distribution. Abbreviations: ALI — Advanced Lung cancer Inflammation Index [1], EPSILoN [2], GPS — Glasgow Prognostic Score [3], GRIm — Gustave Roussy Immune Score [4], Holtzman — score described by Holtzman et. al [5], LIPI — Lung Immune Prognostic Index [6], LIPS-3 — Lung Immuno-oncology Prognostic Score 3 [7], LIPS-4 — Lung Immuno-oncology Prognostic Score 4 [7], mLIPI — Modified Lung Immune Prognostic Index [8], PNI — Prognostic Nutritional Index [9], RMH — Royal Marsden Hospital Prognostic Score [10], SII — Systemic Immune-Inflammation Index [11], and SIPS — Scottish Inflammatory Prognostic Score [12].

Table S2. Sensitivity analysis for the concordance index (c-index) for overall survival and progression-free survival using dichotomized scores.

Scores	Imputation	Complete	Balanced	Non-squamous
**LIPI**				
OS	0.58	0.58	0.58	0.60
PFS	0.57	0.56	0.56	0.59
**mLIPI**				
OS	0.63	0.63	0.64	0.63
PFS	0.60	0.60	0.61	0.61
**SIPS**				
OS	0.59	0.60	0.59	0.60
PFS	0.57	0.58	0.57	0.58
**ALI**				
OS	0.60	0.60	0.59	0.60
PFS	0.57	0.57	0.56	0.57
**EPSILoN**				
OS	0.59	0.59	0.60	0.59
PFS	0.57	0.57	0.57	0.57
**PNI**				
OS	0.60	0.61	0.62	0.61
PFS	0.59	0.59	0.61	0.59
**SII**				
OS	0.57	0.57	0.58	0.58
PFS	0.54	0.54	0.55	0.55
**GRIm**				
OS	0.62	0.62	0.62	0.62
PFS	0.59	0.59	0.59	0.60
**RMH**				
OS	0.63	0.64	0.64	0.64
PFS	0.60	0.61	0.61	0.62
**LIPS-3**				
OS	0.62	0.62	0.64	0.62
PFS	0.59	0.59	0.60	0.59
**LIPS-4**				
OS	0.63	0.63	0.64	0.64
PFS	0.60	0.60	0.60	0.60
**Holtzman**				
OS	0.49	0.49	0.50	0.52
PFS	0.50	0.50	0.51	0.50
**GPS**				
OS	0.61	0.62	0.62	0.62
PFS	0.58	0.59	0.59	0.60

Abbreviations: ALI — Advanced Lung cancer Inflammation Index [1], Balanced — population with the three cohorts balanced in sample size (c-index and HR were the median value of 200 analyses on balanced data), Complete — population with complete data only (removing patients with missing data), EPSILoN [2], GPS — Glasgow Prognostic Score [3], GRIm — Gustave Roussy Immune Score [4], Holtzman — score described by Holtzman et. al [5], Imputation — whole population, including patients with missing scores imputed by the MICE algorithm [13] (main analysis), LIPI — Lung Immune Prognostic Index [6], LIPS-3 — Lung Immuno-oncology Prognostic Score 3 [7], LIPS-4 — Lung Immuno-oncology Prognostic Score 4 [7], mLIPI — Modified Lung Immune Prognostic Index [8], Non-squamous — patients with non-squamous NSCLC, OS — overall survival, PFS — progression-free survival, PNI — Prognostic Nutritional Index [9], RMH — Royal Marsden Hospital Prognostic Score [10], SII — Systemic Immune-Inflammation Index [11], and SIPS — Scottish Inflammatory Prognostic Score [12].

Table S3. Sensitivity analysis for the hazard ratio of the interactions for the difference between cohort 1 and cohort 2 for overall survival and progression-free survival.

Scores	Imputation	Complete	Balanced	Non-squamous
**LIPI**				
OS	0.98	0.98	0.98	0.82
PFS	0.72	0.71	0.69	0.74
**mLIPI**				
OS	0.61	0.62	0.62	0.43
PFS	0.60	0.60	0.59	0.53
**SIPS**				
OS	0.24	0.27	0.23	0.25
PFS	0.20	0.22	0.20	0.21
**ALI**				
OS	1.35	1.31	1.38	1.76
PFS	1.26	1.24	1.24	1.66
**EPSILoN**				
OS	1.31	1.31	1.27	1.65
PFS	1.02	1.01	0.94	1.34
**PNI**				
OS	1.21	1.13	1.22	1.12
PFS	1.07	1.03	1.09	1.28
**SII**				
OS	0.74	0.74	0.72	0.85
PFS	1.22	1.22	1.18	1.25
**GRIm**				
OS	1.03	1.09	1.03	0.87
PFS	0.94	0.95	0.93	0.93
**RMH**				
OS	1.52	1.60	1.49	1.53
PFS	1.39	1.40	1.35	1.49
**LIPS-3**				
OS	0.46	0.46	0.46	0.28
PFS	0.42	0.42	0.41	0.30
**LIPS-4**				
OS	1.02	1.04	1.03	0.68
PFS	0.81	0.80	0.79	0.68
**Holtzman**				
OS	0.60	0.60	0.61	0.71
PFS	0.55	0.55	0.56	0.51
**GPS**				
OS	0.50	0.53	0.51	0.29
PFS	0.62	0.62	0.61	0.36

Abbreviations: ALI — Advanced Lung cancer Inflammation Index [1], Balanced — population with the three cohorts balanced in sample size (c-index and HR were the median value of 200 analyses on balanced data), Complete — population with complete data only (removing patients with missing data), EPSILoN [2], GPS — Glasgow Prognostic Score [3], GRIm — Gustave Roussy Immune Score [4], Holtzman — score described by Holtzman et. al [5], Imputation — whole population, including patients with missing scores imputed by the MICE algorithm [13] (main analysis), LIPI — Lung Immune Prognostic Index [6], LIPS-3 — Lung Immuno-oncology Prognostic Score 3 [7], LIPS-4 — Lung Immuno-oncology Prognostic Score 4 [7], mLIPI — Modified Lung Immune Prognostic Index [8], Non-squamous — patients with non-squamous NSCLC, OS — overall survival, PFS — progression-free survival, PNI — Prognostic Nutritional Index [9], RMH — Royal Marsden Hospital Prognostic Score [10], SII — Systemic Immune-Inflammation Index [11], and SIPS — Scottish Inflammatory Prognostic Score [12].

References

1. Jafri, S. H.; Shi, R.; Mills, G., Advance lung cancer inflammation index (ALI) at diagnosis is a prognostic marker in patients with metastatic non-small cell lung cancer (NSCLC): a retrospective review. *BMC Cancer* **2013,** 13, 158. doi: 10.1186/1471-2407-13-158
2. Prelaj, A.; Rebuzzi, S. E.; Pizzutilo, P.; Bilancia, M.; Montrone, M.; Pesola, F.; Longo, V.; Del Bene, G.; Lapadula, V.; Cassano, F.; Petrillo, P.; Bafunno, D.; Varesano, N.; Lamorgese, V.; Mastrandrea, A.; Ricci, D.; Catino, A.; Galetta, D., EPSILoN: A Prognostic Score Using Clinical and Blood Biomarkers in Advanced Non-Small-cell Lung Cancer Treated With Immunotherapy. *Clin Lung Cancer* **2020,** 21, 365-377.e5. doi: 10.1016/j.cllc.2019.11.017
3. McMillan, D. C., An inflammation-based prognostic score and its role in the nutrition-based management of patients with cancer. *Proc Nutr Soc* **2008,** 67, 257-62. doi: 10.1017/s0029665108007131
4. Bigot, F.; Castanon, E.; Baldini, C.; Hollebecque, A.; Carmona, A.; Postel-Vinay, S.; Angevin, E.; Armand, J. P.; Ribrag, V.; Aspeslagh, S.; Varga, A.; Bahleda, R.; Menis, J.; Gazzah, A.; Michot, J. M.; Marabelle, A.; Soria, J. C.; Massard, C., Prospective validation of a prognostic score for patients in immunotherapy phase I trials: The Gustave Roussy Immune Score (GRIm-Score). *Eur J Cancer* **2017,** 84, 212-218. doi: 10.1016/j.ejca.2017.07.027
5. Holtzman, L.; Moskovitz, M.; Urban, D.; Nechushtan, H.; Keren, S.; Reinhorn, D.; Wollner, M.; Daher, S.; Rottenberg, Y.; Rovitzky, Y.; Shochat, T.; Bar, J.; Dudnik, E., dNLR-Based Score Predicting Overall Survival Benefit for The Addition of Platinum-Based Chemotherapy to Pembrolizumab in Advanced NSCLC With PD-L1 Tumor Proportion Score ≥50%. *Clinical Lung Cancer* **2022,** 23, 122-134. doi: 10.1016/j.cllc.2021.12.006
6. Mezquita, L.; Auclin, E.; Ferrara, R.; Charrier, M.; Remon, J.; Planchard, D.; Ponce, S.; Ares, L. P.; Leroy, L.; Audigier-Valette, C.; Felip, E.; Zerón-Medina, J.; Garrido, P.; Brosseau, S.; Zalcman, G.; Mazieres, J.; Caramela, C.; Lahmar, J.; Adam, J.; Chaput, N.; Soria, J. C.; Besse, B., Association of the Lung Immune Prognostic Index With Immune Checkpoint Inhibitor Outcomes in Patients With Advanced Non-Small Cell Lung Cancer. *JAMA Oncol* **2018,** 4, 351-357. doi: 10.1001/jamaoncol.2017.4771
7. Banna, G. L.; Cortellini, A.; Cortinovis, D. L.; Tiseo, M.; Aerts, J.; Barbieri, F.; Giusti, R.; Bria, E.; Grossi, F.; Pizzutilo, P.; Berardi, R.; Morabito, A.; Genova, C.; Mazzoni, F.; Di Noia, V.; Signorelli, D.; Gelibter, A.; Macerelli, M.; Rastelli, F.; Chiari, R.; Rocco, D.; Gori, S.; De Tursi, M.; Di Marino, P.; Mansueto, G.; Zoratto, F.; Filetti, M.; Montrone, M.; Citarella, F.; Marco, R.; Cantini, L.; Nigro, O.; D'Argento, E.; Buti, S.; Minuti, G.; Landi, L.; Guaitoli, G.; Lo Russo, G.; De Toma, A.; Donisi, C.; Friedlaender, A.; De Giglio, A.; Metro, G.; Porzio, G.; Ficorella, C.; Addeo, A., The lung immuno-oncology prognostic score (LIPS-3): a prognostic classification of patients receiving first-line pembrolizumab for PD-L1 ≥ 50% advanced non-small-cell lung cancer. *ESMO Open* **2021,** 6, 100078. doi: 10.1016/j.esmoop.2021.100078
8. Moor, R.; Roberts, K.; Mason, R.; Ladwa, R.; Lwin, Z.; Hughes, B.; O'Byrne, K., P1.01-119 Modified Lung Immune Prognostic Index (mLIPI) as a Predictive Tool of Nivolumab Outcomes in Advanced NSCLC Patients. *Journal of Thoracic Oncology* **2019,** 14, S408-S409. doi: 10.1016/j.jtho.2019.08.834
9. Onodera, T.; Goseki, N.; Kosaki, G., [Prognostic nutritional index in gastrointestinal surgery of malnourished cancer patients]. *Nihon Geka Gakkai Zasshi* **1984,** 85, 1001-5. doi:
10. Arkenau, H. T.; Olmos, D.; Ang, J. E.; de Bono, J.; Judson, I.; Kaye, S., Clinical outcome and prognostic factors for patients treated within the context of a phase I study: the Royal Marsden Hospital experience. *Br J Cancer* **2008,** 98, 1029-33. doi: 10.1038/sj.bjc.6604218
11. Hu, B.; Yang, X. R.; Xu, Y.; Sun, Y. F.; Sun, C.; Guo, W.; Zhang, X.; Wang, W. M.; Qiu, S. J.; Zhou, J.; Fan, J., Systemic immune-inflammation index predicts prognosis of patients after curative resection for hepatocellular carcinoma. *Clin Cancer Res* **2014,** 20, 6212-22. doi: 10.1158/1078-0432.Ccr-14-0442
12. Stares, M.; Ding, T. E.; Stratton, C.; Thomson, F.; Baxter, M.; Cagney, H.; Cumming, K.; Swan, A.; Ross, F.; Barrie, C.; Maclennan, K.; Campbell, S.; Evans, T.; Tufail, A.; Harrow, S.; Lord, H.; Laird, B.; MacKean, M.; Phillips, I., Biomarkers of systemic inflammation predict survival with first-line immune checkpoint inhibitors in non-small-cell lung cancer. *ESMO Open* **2022,** 7, 100445. doi: 10.1016/j.esmoop.2022.100445
13. The MICE algorithm. Available online: https://cran.r-project.org/web/packages/miceRanger/vignettes/miceAlgorithm.html (accessed on 11 December 2022).
